# The Impact of Biocontrol Agents on the Metabolome of *Penicillium nordicum* Strains and Its Relation to Ochratoxin A Production on Dry-Cured Ham

**DOI:** 10.3390/toxins17050236

**Published:** 2025-05-09

**Authors:** Eva Cebrián, Elia Roncero, João Luz, Mar Rodríguez, Marta Sousa Silva, Carlos Cordeiro, Félix Núñez

**Affiliations:** 1 Food Hygiene and Safety, Meat and Meat Products Research Institute, Faculty of Veterinary Science, University of Extremadura, Avda. de las Ciencias, s/n, 10003 Cáceres, Spain; evcebrianc@unex.es (E.C.); eroncerob@unex.es (E.R.); fnunez@unex.es (F.N.); 2 Laboratório de FT-ICR e Espectrometria de Massa Estrutural, BioISI Biosystems and Integrative Sciences Institute, Faculdade de Ciências da Universidade de Lisboa, Campo Grande, 1749-016 Lisbon, Portugal; jmluz@fc.ul.pt (J.L.); mfsilva@ciencias.ulisboa.pt (M.S.S.); cacordeiro@ciencias.ulisboa.pt (C.C.)

**Keywords:** ochratoxigenic *P. nordicum*, intra-species differences, *D. hansenii*, *S. xylosus*, *P. chrysogenum*, untargeted metabolomics, dry-cured ham

## Abstract

Throughout the process of dry-cured ham, moulds such as *P. nordicum*, a producer of ochratoxin A (OTA), grow on its surface. The use of combined biocontrol agents (BCAs) is a promising strategy for controlling this hazard. The goal of this study is to assess the effect of *D. hansenii*, *S. xylosus*, and *P. chrysogenum* as BCAs on the metabolome of two strains of *P. nordicum* and to understand the differences between both strains. Each ochratoxigenic strain was inoculated both individually and in combination with the BCAs onto ham for 30 days under the environmental conditions experienced during traditional ripening. Untargeted metabolomics was performed through mass spectrometry using a Q-Exactive Plus Orbitrap. The BCAs caused alterations in the metabolomes of both ochratoxigenic moulds, mainly in phenylalanine catabolism and the valine, leucine, and isoleucine biosynthesis pathways, although with some differences. In the absence of the BCAs, the metabolomes of both types of *P. nordicum* were globally changed, despite these being moulds of the same species. In conclusion, these data help us to understand the differences between OTA-producing strains in dry-cured ham and confirm the need to demonstrate the efficacy of BCAs against a wide range of toxigenic moulds before they can be used to minimise OTA contamination in the meat industry.

## 1. Introduction

The processing of dry-cured ham has well-defined stages: pre-salting and salting, where curing salts are added to facilitate a reduction in water activity to inhibit altering and pathogenic microorganisms; post-salting, a stage in which the salt and nitrate contents inside the hams are balanced; and finally, the drying–ripening stage, where the enzymatic reactions necessary for the development of the characteristic aroma and flavour of this meat product take place [1,2]. In this last phase, environmental conditions support the growth of microorganisms on the surface, mainly moulds. Fungal growth favourably impacts the sensory characteristics of the final product [3,4,5]. However, uncontrolled growth can lead to alterations in the appearance and aroma of the product [6] or to the accumulation of mycotoxins, a serious food safety issue. Ochratoxin A (OTA) is the mycotoxin most frequently detected in dry-cured meat products [7,8,9], with *Penicillium nordicum* being the most common ochratoxigenic mould isolated from these foods [10,11,12,13,14]. OTA is rapidly absorbed and distributed in humans, but it is eliminated and excreted slowly [15], leading to its potential accumulation in the bodies of consumers. This mycotoxin is nephrotoxic, hepatotoxic, immunotoxin, neurotoxic, and teratogenic [16,17], and it is considered to belong to category 2B as a possible human carcinogen by the International Agency for Research on Cancer [18,19]. However, some studies have suggested that it should be considered for reclassification into category 2A as a probable human carcinogen [20,21]. Therefore, there is a need to identify strategies for controlling the growth of unwanted moulds in dry-cured ham without disturbing beneficial microbiota. For this purpose, the use of microorganisms that commonly grow on this meat product as biocontrol agents (BCAs) is a promising strategy. Previous studies have shown that yeasts such as *Debaryomyces hansenii*, Gram-positive and catalase-positive cocci such as *Staphylococcus xylosus*, and non-toxigenic moulds such as *Penicillium chrysogenum* are effective in reducing OTA contamination in dry-cured ham [22,23,24,25]. These BCAs trigger changes in the proteome and in several metabolic processes which impact the synthesis of secondary metabolites such as OTA, and the combination of different BCAs can have a synergistic effect and lead to a better result [24].

On the other hand, BCAs act differently depending on the specific mould they encounter [26,27], and strains of OTA-producing moulds from the same species may behave differently [28,29]. Therefore, it is important to study the behaviour of different strains of toxigenic mould when confronted with BCAs on a meat substrate. 

Recent advancements in omics technologies have significantly enhanced the study of food microbiology [30,31]. Metabolomic analyses allow for the identification of metabolites and the generation of a metabolic profile [32] to unveil the relevant metabolites depleted or overproduced in diverse environmental, genetic, pathological, and developmental circumstances [33]. Thus, it is possible to link particular metabolic profiles to the synthesis of toxins or other relevant compounds such as OTA [34]. For this reason, metabolomics allows the changes in mould metabolism caused by BCAs to be elucidated [27]. Identification of the pathways and metabolites involved could help to understand the behaviour of each toxigenic strain.

The main goal of this work is to assess how the metabolic pathways of two *P. nordicum* strains are affected by interaction with BCAs and to understand the difference between both strains involved in OTA production in dry-cured ham.

## 2. Results

### 2.1. The Effect of BCAs on the Metabolome of OTA-Producer Moulds

The combined use of *D. hansenii*, *S. xylosus*, and *P. chrysogenum* resulted in a significant decrease in the OTA yield from Pn15 and Pn856 as assessed using targeted metabolomics in dry-cured ham at 30 days of incubation [24]. Hence, untargeted metabolomics was performed for each *P. nordicum* growth condition (Pn15 vs. Pn15 + BCAs and Pn856 vs. Pn856 + BCAs). Table 1 shows the qualitative and quantitative changes in this metabolite’s abundance for each of the comparisons.

**Figure 1:**
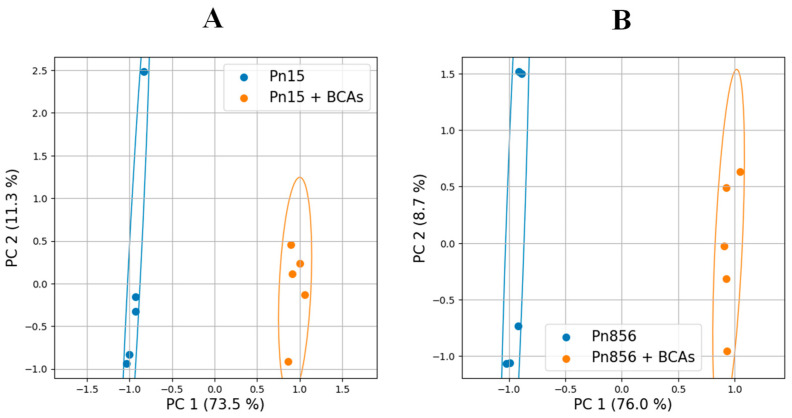
Score plots of the multivariate Principal Component Analysis (PCA) of the metabolome of the batches inoculated with *P. nordicum* FHSCC 15 (**A**) and *P. nordicum* BFE 856 (**B**) after 30 days in dry-cured ham. Pn15: Control samples inoculated with *P. nordicum* FHSCC 15; Pn15 + BCAs: samples inoculated with *P. nordicum* FHSCC 15, *D. hansenii* FHSCC 253H, *S. xylosus* FHSCC Sx8, and *P. chrysogenum* FHSCC Pg222; Pn856: control samples inoculated with *P. nordicum* BFE 856; Pn856 + BCAs: samples inoculated with *P. nordicum* BFE 856, *D. hansenii* FHSCC 253H, *S. xylosus* FHSCC Sx8, and *P. chrysogenum* FHSCC Pg222. The total variance explained is above 80%.

Figure 1 and Figure S1 show the Principal Component Analysis (PCA) and heat maps, respectively, where a clear difference was observed between the batches inoculated only with the OTA-producer moulds (Pn15; Pn856) and the samples also inoculated with the BCAs (Pn15 + BCAs; Pn856 + BCAs). This means that the BCAs caused relevant variations in the metabolomic profiles of the two ochratoxigenic moulds.

**Figure 2:**
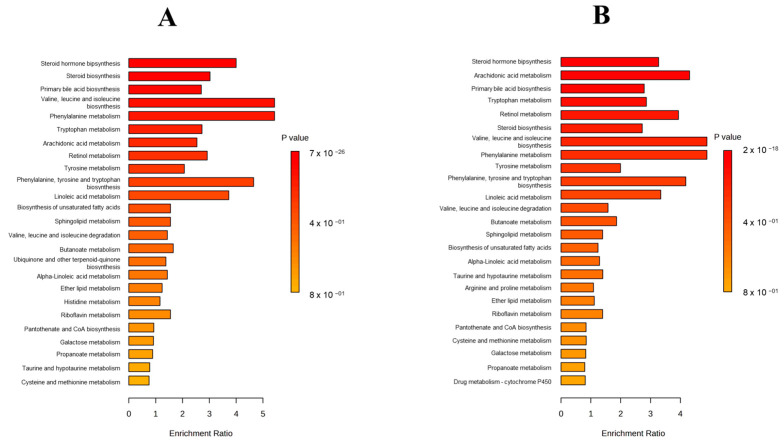
Metabolite Set Enrichment Analysis (MSEA) (top 25) of *P. nordicum* FHSCC 15 (**A**) and *P. nordicum* BFE 856 (**B**) metabolomes inoculated with *D. hansenii* FHSCC 253H, *S. xylosus* FHSCC Sx8, and *P. chrysogenum* FHSCC Pg222 after 30 days in dry-cured ham.

In Figure 2, a Metabolite Set Enrichment Analysis (MSEA) is shown, in which it was observed that the two most significant altered pathways in both ochratoxigenic moulds were valine, leucine, and isoleucine biosynthesis and phenylalanine metabolism. In contrast, the third most significant pathway in each of the moulds was different, with this being phenylalanine, tyrosine, and tryptophan biosynthesis in Pn15 and the arachidonic acid metabolism pathway in Pn856 (Figure 2).

Therefore, the alterations in the abundance of the metabolites in these pathways were investigated, and the results obtained are shown in Table 2 and Table 3. In the phenylalanine metabolism pathway, five compounds were altered in Pn15. Specifically, one of them was detected only in the batch inoculated exclusively with the OTA-producer mould, three were only detected in the ham samples inoculated with the BCAs, and one was increased in its abundance due to their presence. In Pn856, six metabolites were only found in the samples inoculated with the OTA-producer mould, and six were increased in the presence of the BCAs (Table 2).

Another significant pathway for both OTA-producer moulds was valine, leucine, and isoleucine biosynthesis. In this pathway, only one increased metabolite was found in the control batch with Pn15. One metabolite was exclusively detected in the batch inoculated with Pn856, and the abundance of two metabolites was raised in the samples inoculated with the BCAs (Table 2).

On the other hand, in the Pn15 study, the phenylalanine, tyrosine, and tryptophan biosynthesis pathway had a high enrichment ratio, showing three altered metabolites. One of them was only found in the samples inoculated with Pn15, and two were increased by the presence of BCAs (Table 3).

Arachidonic acid metabolism was a very important pathway in the study with Pn856. The action of the BCAs was clearly seen, as 7 metabolites were detected only in the batch inoculated with them, and 12 were increased in their presence (Table 3).

**Figure 3:**
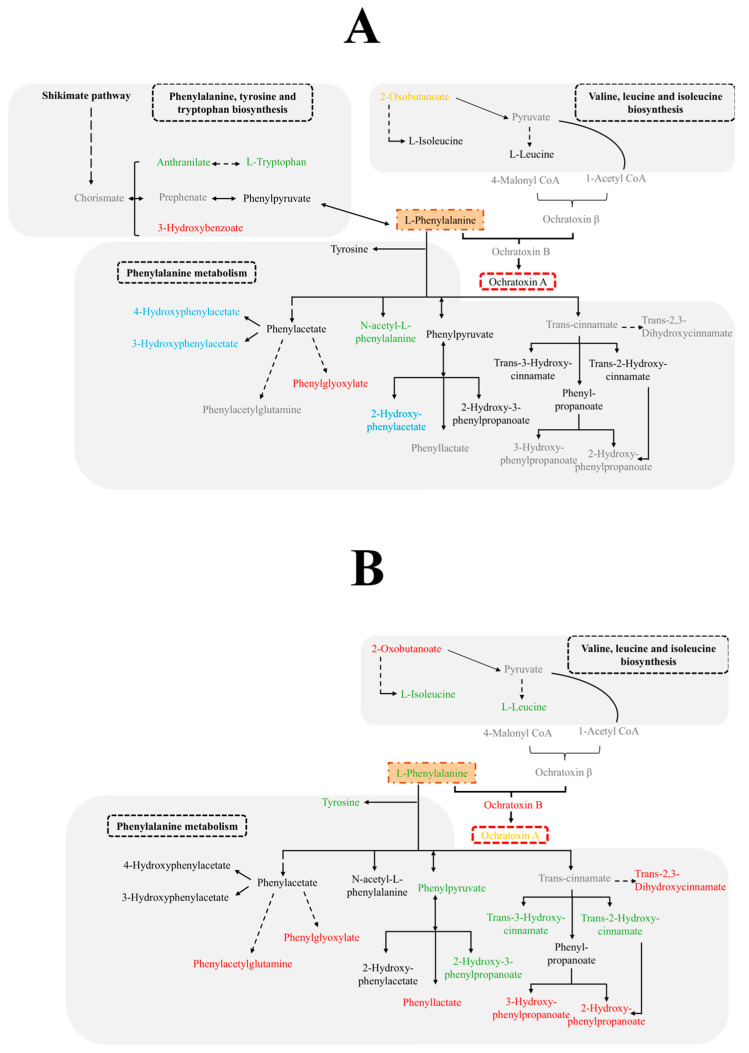
Metabolic pathways affected by *D. hansenii* FHSCC 253H, *S. xylosus* FHSCC Sx8, and *P. chrysogenum* FHSCC Pg222 in the metabolomes of *P. nordicum* FHSCC 15 (**A**) and *P. nordicum* BFE 856 (**B**). Metabolites not detected (grey colour); metabolites without significant differences (black colour); metabolites exclusively detected in the samples inoculated only with *P. nordicum* (red colour); metabolites only detected in the presence of the BCAs (blue); metabolites increased in the presence of the BCAs (green colour); metabolites decreased in the presence of the BCAs (orange colour).

On the other hand, compounds from the OTA biosynthesis pathway were also found, such as Ochratoxinβ, detected exclusively in the samples inoculated with Pn856, and OTA, decreased in this ochratoxigenic mould by the action of the BCAs, as can be seen in Figure 3.

### 2.2. Metabolomics Changes Between Both Strains of OTA-Producing P. nordicum

**Figure 4:**
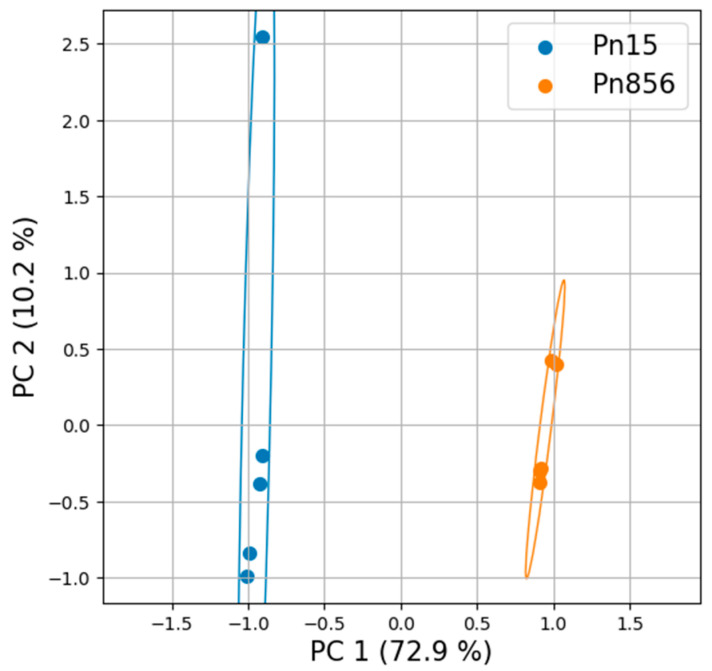
Univariant Principal Component Analysis (PCA) of the metabolome of the batches inoculated with *P. nordicum* FHSCC 15 (Pn15) and *P. nordicum* BFE 856 (Pn856) in dry-cured ham. The total variance explained is above 80%.

Since significant differences were observed in the way that the BCAs acted against each *P. nordicum* strain, the metabolomic profiles of these strains in the absence of the BCAs (Pn15 vs. Pn856) were compared to find out how each strain of the same species behaved. In this respect, the PCA and the Hierarchical Clustering Analysis (HCA) represented in Figure 4 and Figure S2 showed a clear difference between them.

The quantitative and qualitative changes in the abundance of metabolites were studied. In this respect, 1140 compounds were found only in the batch inoculated with Pn15, while 1222 were found only in the batch inoculated with Pn856, and 4020 were common between both toxigenic moulds.

**Figure 5:**
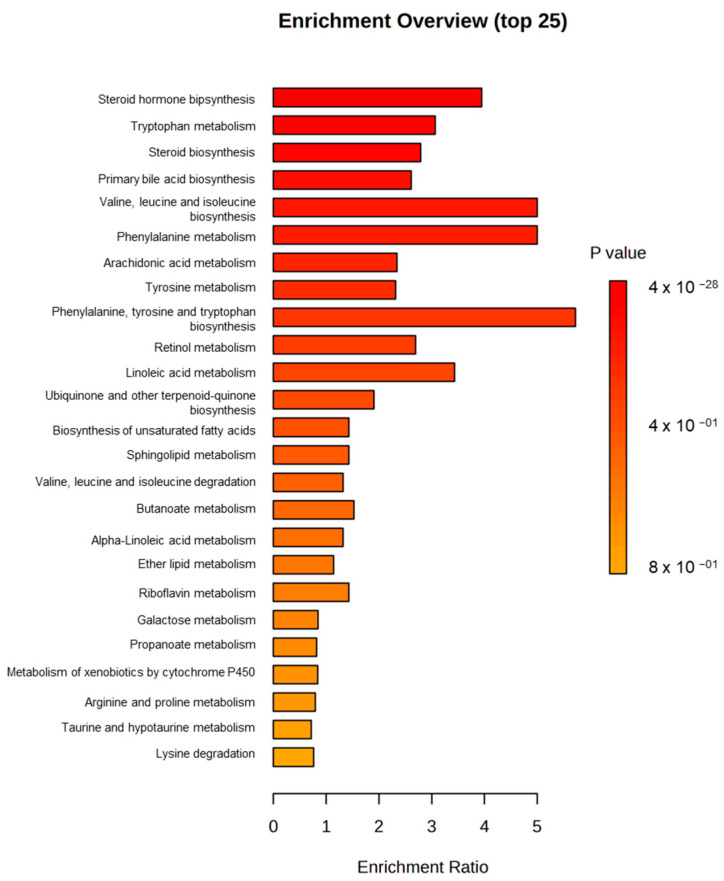
Metabolite Set Enrichment Analysis (MSEA) (top 25) of *P. nordicum* FHSCC 15 and *P. nordicum* BFE 856 after 30 days in dry-cured ham.

These differences were revealed by the MSEA shown in Figure 5, where phenylalanine, tyrosine, and tryptophan biosynthesis; phenylalanine metabolism; and valine, leucine, and isoleucine biosynthesis showed the highest changes. Therefore, the metabolites involved in these pathways were analysed, and the result is shown in Table 4. In phenylalanine, tyrosine, and tryptophan biosynthesis, one metabolite was only found in the samples inoculated with Pn856 and another in the batch inoculated only with Pn15. Relevant differences were also found in the phenylalanine metabolism pathway, with eight compounds detected only in the Pn856 batch and one in the Pn15 batch. On the other hand, in the valine, leucine, and isoleucine biosynthesis pathway, (S)-3-Methyl-2-oxopentanoate was reduced in the batch inoculated with Pn856.

**Figure 6:**
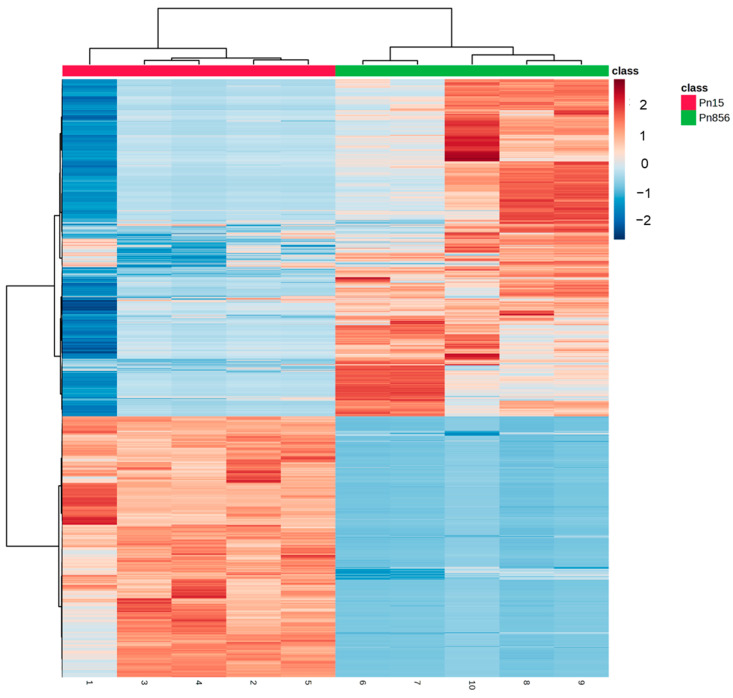
Heat map of significant compounds in *P. nordicum* FHSCC 15 (Pn15, red colour) and *P. nordicum* BFE 856 (Pn856, green colour) after 30 days in dry-cured ham.

The most significant compounds detected in each batch reveal a clear difference in the distribution of the metabolites, as shown in the heat map in Figure 6. Therefore, an MSEA of the metabolites increased in each mould was carried out to identify which pathways were more relevant in each strain.

**Figure 7:**
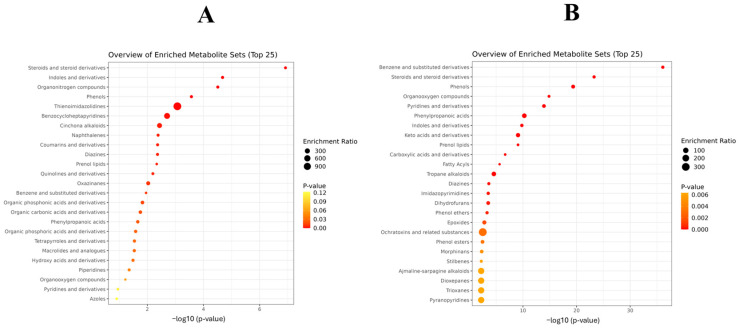
Metabolite Set Enrichment Analysis (MSEA) (top 25) of the compounds increased by *P. nordicum* FHSCC 15 (**A**) and *P. nordicum* BFE 856 (**B**) after 30 days in dry-cured ham.

When performing the MSEA with the compounds increased in the batch inoculated with Pn856, it was found that one of the pathways with the highest enrichment ratio was ochratoxins and related substances, as can be seen in Figure 7. However, when studying the metabolites increased in the batch inoculated with Pn15, it was found that one of the most relevant pathways was that for thienoimidazolidines (Figure 7).

## 3. Discussion

The production of OTA by *P. nordicum* throughout the ripening of dry-cured ham involves a potential hazard for consumers. Therefore, the use of BCAs to prevent this hazard is becoming more popular. The combined use of *D. hansenii*, *S. xylosus,* and *P. chrysogenum* effectively controlled the OTA production during the ripening of dry-cured ham [24]. Changes in proteins related to both cell wall integrity and OTA synthesis are one of the main ways in which both *D. hansenii* and *S. xylosus*, used as individual BCAs, act to decrease this mycotoxin [35,36]. On the other hand, the combination of these microorganisms causes an alteration in the metabolome of several OTA-producer moulds without producing toxic secondary metabolites [27]. However, to the best of our knowledge, the impact of *D. hansenii* and *S. xylosus* together with *P. chrysogenum* on the metabolome of OTA-producing moulds has not yet been studied.

### 3.1. The Effect of the BCAs on the Metabolome of the Ochratoxigenic Moulds

In this respect, the BCAs caused a clear alteration in the metabolome of the two *P. nordicum* strains studied (Figure 1 and Figure S1). Concretely, phenylalanine metabolism and valine, leucine, and isoleucine biosynthesis are among the main pathways altered in both ochratoxigenic moulds (Figure 2 and Figure 3).

L-phenylalanine is essential to OTA biosynthesis in providing its molecular scaffold [37] (Figure 4). The presence of *D. hansenii*, *S. xylosus,* and *P. chrysogenum* increased the abundance of this amino acid but also that of compounds derived from the L-phenylalanine catabolism in Pn856. In addition, 80% of the metabolites detected in the L-phenylalanine metabolism, in comparison with Pn15, were also increased in the presence of the BCAs, although they did not cause a change in the abundance of L-phenylalanine. This could explain the reduction in OTA observed in a previous study [24]; given that L-phenylalanine is more actively metabolised through other pathways in the presence of the BCAs, there will be a smaller amount available for OTA biosynthesis. These results agree with those obtained by Cebrián et al. [27], who also found that *D. hansenii* and *S. xylosus* increased the catabolites derived from the L-phenylalanine pathways in both strains of *P. nordicum*.

*D. hansenii*, *S. xylosus*, and *P. chrysogenum* also altered the biosynthesis of valine, leucine, and isoleucine in both strains. In particular, the metabolite 2-Oxobutanoate had a higher abundance in the control batches inoculated without the BCAs. This metabolite is involved in the synthesis of Acetyl-CoA (https://www.genome.jp/pathway/sde00290) (accessed on 11 April 2025) (Figure 4), which is itself an OTA precursor since a polyketide synthase (PKS) combines Acetyl-CoA and 4-Malonyl-CoA to synthetise 7-Methylmellein, which is then oxidised into ochratoxin β [38,39]. In our study, no changes in Acetyl-CoA abundance were detected in the presence of the BCAs. However, in Pn856, the BCAs caused an increase in the abundance of L-Isoleucine, derived from 2-Oxobutanoate. Hence, the metabolism of 2-Oxobutanoate is oriented towards the production of this amino acid (Figure 3) rather than Acetyl-CoA and therefore OTA.

On the other hand, the changes in the pathways caused by the BCAs are different in each strain. For phenylalanine, tyrosine, and tryptophan biosynthesis, the modifications are more relevant in Pn15 than in Pn856 (Figure 2). In this sense, an increase in the abundance of anthranilate was detected when Pn15 was inoculated together with the BCAs. Anthranilate is produced from Chorismate (which was not detected in our study), which in turn is involved in the shikimic acid pathway, which leads to the synthesis of Prephenate, L-Phenylalanine, and finally OTA [40,41] (Figure 3). These facts could indicate that Chorismate is used to form Anthranilate and L-Tryptophan, instead of the OTA synthesis pathway being followed (https://www.genome.jp/pathway/map00400) (accessed on 11 April 2025).

Otherwise, the arachidonic acid metabolism pathway was more relevant in Pn856 (Figure 3). The BCAs caused an increase in the abundance of metabolites related to this pathway, which led to oxylipin formation [42,43]. Oxylipins are recognised as key metabolites involved in regulating mycotoxin production, conidiogenesis, and sclerotia development in fungi [44,45,46]. In addition, a previous study showed that *D. hansenii* and *S. xylosus* caused a decrease in the production of oxylipins derived from linoleic acid metabolism in both *P. nordicum* strains after co-inoculation at 14 days in dry-cured ham [27]. This contrary effect may be due to several factors, such as the addition of *P. chrysogenum* as another BCA; the temperature, which gradually increased from 12 to 18 °C; and/or the incubation time, which in this study was 30 days. Nevertheless, mycotoxin production may be regulated by the balance between different oxylipins, with some stimulating and others inhibiting mycotoxin synthesis [47]. Therefore, it cannot be ruled out that the higher abundance of arachidonic-acid-derived oxylipins may have been related to the decrease in the production of OTA by Pn856, and further studies are needed to establish this connection.

### 3.2. Metabolomics Changes Between Both OTA-Producing P. nordicum Strains

The strains of *P. nordicum* in the absence of the BCAs had completely different metabolomic profiles (Figure 3). Specifically, the main disparities were observed in phenylalanine, tyrosine, and tryptophan biosynthesis; phenylalanine metabolism; and valine, leucine, and isoleucine biosynthesis (Figure 5). The most significant differences were found in phenylalanine metabolism, where 89% of the significant compounds were found only in Pn856. This could be related to the fact that this strain is able to produce higher concentrations of OTA in dry-cured ham [24], and since L-phenylalanine is a precursor of OTA [37], its metabolism should be increased. In the same way, it could also be explained that one of the most relevant pathways for this strain is that for ochratoxins and related substances (Figure 7). Furthermore, this is in line with previous studies showing that OTA production is dependent on the strain of ochratoxigenic mould that produces it [28]. The thienoimidazolidine pathway appears to have significant relevance in Pn15. Although these compounds have an antimicrobial effect, to the best of our knowledge, there is no existing information that helps us to understand its role in ochratoxigenic moulds, and further research will be necessary.

It has been shown that different strains of *P. nordicum* can exhibit differences in their growth, toxin production, gene expression, or proteomic profiles when they are grown on a variety of media or treated with bioprotective agents [28,35]. This intra-species variability is evidenced by the different metabolomic profiles detected.

## 4. Conclusions

The application of *D. hansenii* FHSCC 253H, *S. xylosus* FHSCC Sx8, and *P. chrysogenum* FHSCC Pg222 as a mixed protective culture caused alterations in the metabolomes of both strains of *P. nordicum,* although not in the same way. Furthermore, the metabolomes of *P. nordicum* FHSCC 15 and *P. nordicum* BFE 856 in the absence of the BCAs are also different. These data will be helpful not only for revealing the differences between strains involved in OTA contamination in dry-cured ham but also for validating the use of microorganisms as BCAs and demonstrating their efficacy against a wide variety of ochratoxigenic moulds. This will prove useful for designing effective preventive measures to minimise OTA contamination throughout the ripening of dry-cured ham.

## 5. Materials and Methods

### 5.1. Microorganisms

Two ochratoxin A-producing moulds were used for this study: *P. nordicum* FHSCC 15 (Pn15) from the Food Hygiene and Safety Culture Collection at the University of Extremadura (Cáceres, Spain) and *P. nordicum* BFE 856 (Pn856) from the Federal Research Centre for Nutrition and Food (Kulmbach, Germany). *D. hansenii* FHSCC 253H, *S. xylosus* FHSCC Sx8, and *P. chrysogenum* FHSCC Pg222 from the Food Hygiene and Safety Culture Collection at the University of Extremadura were utilised as the BCAs. All of the microorganisms used were isolated from dry-cured ham. Previous studies have demonstrated the bioprotective potential of these microorganisms [24].

### 5.2. The Experimental Setting

The microorganisms were inoculated onto ham pieces at the final post-salting stage as described by Cebrián et al. [24], establishing two batches for each strain of *P. nordicum*. The control batch was inoculated only with each ochratoxigenic mould (Pn15; Pn856), and another batch was inoculated with the ochratoxigenic mould together with the three BCAs (Pn15 + BCAs; Pn856 + BCAs).

Each portion of ham was inoculated through immersion for 10 s in 200 mL of a suspension of the appropriate microorganisms depending on the batch at a concentration of 10^6^ cfu/mL or 10^6^ spores/mL. The inoculated ham pieces were incubated in sterile containers for 30 days, increasing the temperature progressively from 12 °C to 18 °C. The relative humidity was maintained between 85 and 86% to simulate the water loss that occurs during processing, as described by Cebrián et al. [24].

### 5.3. Metabolite Extraction

After 30 days of incubation, 5 g of each portion of dry-cured ham was taken. The extraction of the total metabolites was carried out using the QuEChERS methodology according to Cebrián et al. [27]. Briefly, the extraction method consisted of extracting all of the metabolites with water and acetonitrile (Fisher Scientific, Waltham, MA, USA) acidified with 0.1% acetic acid (*v*/*v*; Fisher Scientific) and phase partitioning using NaCl (Fisher Scientific) and MgSO_4_ (Scharlab, S.L). Next, the mix was shaken by hand and centrifuged for 5 min at 2630× *g* Digtor 21R centrifuge (Ortoalresa, Madrid, Spain), and an aliquot of 1 mL of the supernatant was collected and kept at −20 °C until analysis.

### 5.4. Untargeted Metabolomics Analysis

All of the compounds obtained from each sample were analysed using a Dionex UltiMate 3000 RSLC system coupled with a Q-Exactive high-resolution mass spectrometer (Thermo Fisher Scientific, USA), following the methodology previously described by Cebrián et al. [27]. For every analysis, an ESI source (HESI II, Thermo Fischer Scientific) operating in positive ion mode (ESI+) was used.

The total samples, except the Pool IDs, were analysed using the full-scan method in the 53.4 to 800 *m*/*z* range using a 70.000 Full Width at Half Maximum (FWHM) resolution set at 200 *m*/*z*. Data-Dependent Acquisition (DDA) Top 5 was used to analyse the Pool IDs.

The data were acquired using Compound Discoverer 3.3 software (Thermo Fisher Scientific). For this purpose, a mass trace was generated for each ionised and detected metabolite, followed by RT alignment, using quality control samples and compound using multiple databases (ChemSpider, mzCloud, Natural Products Atlas 2020_06, and an endogenous metabolite database with 4400 compounds).

The data processing and analysis were performed using in-house software written in the Python programming language and developed by the FT-ICR-MS Lisboa group of Cebrián et al. [27].

Spectral alignment was carried out using mass and retention time data, allowing for maximum deviations of 2 ppm and 0.5 min, respectively. Subsequently, the data were re-annotated through the Human Metabolome Database [48], with the corresponding KEGG ID information incorporated [49].

In addition, for the MSEA and creation of the heat maps, the commercial databases, KEGG, and MetaboAnalyst 6.0 (https://www.metaboanalyst.ca/MetaboAnalyst/) (accessed on 11 April 2025) were used.

### 5.5. The Statistical Analysis

The statistical analysis of the metabolomics data was conducted using the custom Python-based software previously described. Missing values were imputed by assigning 1/5 of the lowest detected intensity within each sample, and data normalisation was performed relative to the total signal intensity. To prevent the dominance of highly abundant metabolites, Pareto scaling and a logarithmic transformation (base 2) were applied. Univariate comparisons were carried out using the Mann–Whitney test. Significant inter-group variations in the metabolic profiles were detected when the Log_2_ fold change was >1 or <−1, and the *p*-values were <0.05. PCA and HCA were used as multivariate statistical analysis techniques. Both the univariate and multivariate analyses were conducted using the Python-based software. Additionally, group comparisons were visualised using the cluster analysis heat maps generated using MetaboAnalyst 6.0.

## Figures and Tables

**Table 1:** The number of metabolites with an altered abundance in each comparison of the metabolome in the control (Pn15 or Pn856) against the BCAs (Pn15 + BCAs or Pn856 + BCAs) after 30 days in dry-cured ham. Pn15: Control samples inoculated with *P. nordicum* FHSCC 15; Pn15 + BCAs: samples inoculated with *P. nordicum* FHSCC 15, *D. hansenii* FHSCC 253H, *S. xylosus* FHSCC Sx8, and *P. chrysogenum* FHSCC Pg222; Pn856: control samples inoculated with *P. nordicum* BFE 856; Pn856 + BCAs: samples inoculated with *P. nordicum* BFE 856, *D. hansenii* FHSCC 253H, *S. xylosus* FHSCC Sx8, and *P. chrysogenum* FHSCC Pg222.

Comparative Analysis	Total	Only Detected in Control	Only Detected in BCAs	Common
Pn15 vs. Pn15 + BCAs	5915	1220	755	3940
Pn856 vs. Pn856 + BCAs	6655	1176	1440	4039

**Table 2:** Variations in the relative abundance of compounds related to the two most significant pathways of *P. nordicum* FHSCC 15 (Pn15) and *P. nordicum* BFE 856 (Pn856) caused by *D. hansenii* FHSCC 253H, *S. xylosus* FHSCC Sx8, and *P. chrysogenum* FHSCC Pg222 after 30 days in dry-cured ham. The KEGG ID for each metabolite is also presented.

Phenylalanine Metabolism			
		Log_2_ fold change (control/BCAs)	
Metabolite	KEGG ID	Pn15	Pn856
Phenylglyoxylate	C02137	Only in Pn15 ^a^	Only in Pn856 ^b^
4-Hydroxyphenylacetate	C00642	Only in BCAs ^c^	n.s
3-Hydroxyphenylacetate	C05593	Only in BCAs	n.s
2-Hydroxyphenylacetate	C05852	Only in BCAs	n.s
N-Acetyl-L-phenylalanine	C03519	−1.553	n.s
2-Hydroxyphenylpropanoate	C01198	n.d	Only in Pn856
Phenylacetylglutamine	C04148	n.d	Only in Pn856
Phenyllactate	C05607	n.d	Only in Pn856
3-Hydroxyphenylpropanoate	C11457	n.d	Only in Pn856
trans-2,3-Dihydroxycinnamate	C12623	n.d	Only in Pn856
L-Phenylalanine	C00079	n.s	−2.428
L-Tyrosine	C00082	n.s	−1.730
Phenylpyruvate	C00166	n.s	−3.679
trans-2-Hydroxycinnamate	C01772	n.s	−3.679
2-Hydroxy-3-phenylpropenoate	C02763	n.s	−3.679
trans-3-Hydroxycinnamate	C12621	n.s	−3.679
**Valine, leucine, and isoleucine biosynthesis**			
		Log_2_ fold change (control/BCAs)	
Metabolite	KEGG ID	Pn15	Pn856
2-Oxobutanoate	C00109	6.791	Only in Pn856
L-Leucine	C00123	n.s	−2.050
L-Isoleucine	C00407	n.s	−2.050

^a^ Only in Pn15: a metabolite exclusively detected in the batch inoculated only with *P. nordicum* FHSCC 15. ^b^ Only in Pn856: a metabolite exclusively detected in the batch inoculated only with *P. nordicum* BFE 856. ^c^ Only in BCAs: a metabolite exclusively detected in the batch inoculated with the ochratoxigenic moulds and *D. hansenii* FHSCC 253H, *S. xylosus* FHSCC Sx8, and *P. chrysogenum* FHSCC Pg222. n.s: no significant differences (*p* > 0.05; log_2_FC between 1 and −1). n.d: not detected.

**Table 3:** Variations in the relative abundance of compounds related to phenylalanine, tyrosine, and tryptophan biosynthesis pathways in *P. nordicum* FHSCC 15 (Pn15) and arachidonic acid metabolism of *P. nordicum* BFE 856 (Pn856) caused by *D. hansenii* FHSCC 253H, *S. xylosus* FHSCC Sx8, and *P. chrysogenum* FHSCC Pg222 after 30 days in dry-cured ham. The KEGG ID for each metabolite is also presented.

Phenylalanine, Tyrosine, and Tryptophan Biosynthesis		
		Log_2_ fold change (control/BCAs)
Metabolite	KEGG ID	Pn15
3-Hydroxybenzoate	C00587	Only in Pn15 ^a^
L-tryptophan	C00078	−1.622
Anthranilate	C00108	−1.584
**Arachidonic acid metabolism**		
		Log_2_ fold change (control/BCAs)
Metabolite	KEGG ID	Pn856
Prostaglandin F2alpha	C00639	Only in BCAs ^b^
11-epi-Prostaglandin F2alpha	C05959	Only in BCAs
9,11,15-Trihydroxy-prosta-5,13-dien-1-oic acid	C13809	Only in BCAs
11,12,15-THETA	C14782	Only in BCAs
Trioxilin A3	C14809	Only in BCAs
Trioxilin B3	C14811	Only in BCAs
11,14,15-THETA	C14814	Only in BCAs
Phosphatidylcholine	C00157	−7.447
(15S)-15-Hydroxy-5,8,11-cis-13-trans-eicosatetraenoate	C04742	−1.886
5(S)-HETE	C04805	−1.886
20-HETE	C14748	−1.886
19(S)-HETE	C14749	−1.886
5,6-EET	C14768	−1.886
8,9-EET	C14769	−1.886
14,15-EET	C14771	−1.886
8(S)-HETE	C14776	−1.886
12(S)-HETE	C14777	−1.886
16(R)-HETE	C14778	−1.886
11(R)-HETE	C14780	−1.886

^a^ Only in Pn15: a metabolite exclusively detected in the batch inoculated only with *P. nordicum* FHSCC 15. ^b^ Only in BCAs: a metabolite exclusively detected in the batch inoculated with the ochratoxigenic moulds and *D. hansenii* FHSCC 253H, *S. xylosus* FHSCC Sx8, and *P. chrysogenum* FHSCC Pg222.

**Table 4:** Variations in the relative abundance of metabolites associated with the three most relevant pathways in *P. nordicum* FHSCC 15 (Pn15) and *P. nordicum* BFE 856 (Pn856) after 30 days in dry-cured ham. The KEGG ID for each metabolite is also presented.

Phenylalanine, Tyrosine, and Tryptophan Biosynthesis		
Metabolite	KEGG ID	Log_2_ fold change (Pn15/Pn856)
4-Hydroxyphenylpyruvate	C00642	Only in Pn856 ^a^
3- Hydroxybenzoate	C00587	Only in Pn15 ^b^
**Phenylalanine metabolism**		
Metabolite	KEGG ID	Log_2_ fold change (Pn15/Pn856)
4-Hydroxyphenylpyruvate	C00642	Only in Pn856
3-Hydroxyphenylpyruvate	C05593	Only in Pn856
2-Hydroxyphenylacetate	C05852	Only in Pn856
Phenyllactate	C05607	Only in Pn856
Trans-2,3-Dihydroxycinnamate	C12623	Only in Pn856
2-Hydroxyphnylpropanoate	C01198	Only in Pn856
3-Hydroxyphnylpropanoate	C11457	Only in Pn856
Phenylacetylglutamine	C04148	Only in Pn856
Phenylpropanoate	C05629	Only in Pn15
**Valine, leucine, and isoleucine biosynthesis**		
Metabolite	KEGG ID	Log_2_ fold change (Pn15/Pn856)
(S)-3-Methyl-2-oxopentanoate	C00671	−2.74

^a^ Only in Pn856: This metabolite was exclusively detected in the batch inoculated only with *P. nordicum* BFE 856. ^b^ Only in Pn15: This metabolite was exclusively detected in the batch inoculated only with *P. nordicum* FHSCC 15.

## Data Availability

The original contributions presented in this study are included in the article/Supplementary Material. Further inquiries can be directed to the corresponding author(s).

## Supplementary Materials

The following supporting information can be downloaded at https://www.mdpi.com/article/10.3390/toxins17050236/s1. Figure S1: Metabolomic heat maps for P. nordicum FHSCC 15 (A) and P. nordicum BFE 856 (B) after 30 days in dry-cured ham; Figure S2: Hierarchical Clustering Analysis (HCA) univariant analysis of metabolome of the batches inoculated with *P. nordicum* FHSCC 15 (Pn15) and *P. nordicum* BFE 856 (Pn856) in dry-cured ham.
