# Supplementary material for: The Impact of Biocontrol Agents on the Metabolome of Penicillium nordicum Strains and Its Relation to Ochratoxin A Production on Dry-Cured Ham
Source: Toxins (Basel). 2025 May 9;17(5):236. doi: 10.3390/toxins17050236 (PMC12115904; doi:10.3390/toxins17050236)
Supplement: Supplementary file 1 [file toxins-17-00236-s001.zip › toxins-3625959-supplementary.pdf]

Supplementary Materials: The Impact of Biocontrol Agents on the Metabolome of Penicillium nordicum Strains and Its Relation to Ochratoxin A Production on Dry-Cured Ham

Eva Cebrián, Elia Roncero [1], João Luz, Mar Rodríguez, Marta Sousa Silva, Carlos Cordeiro and Félix Núñez

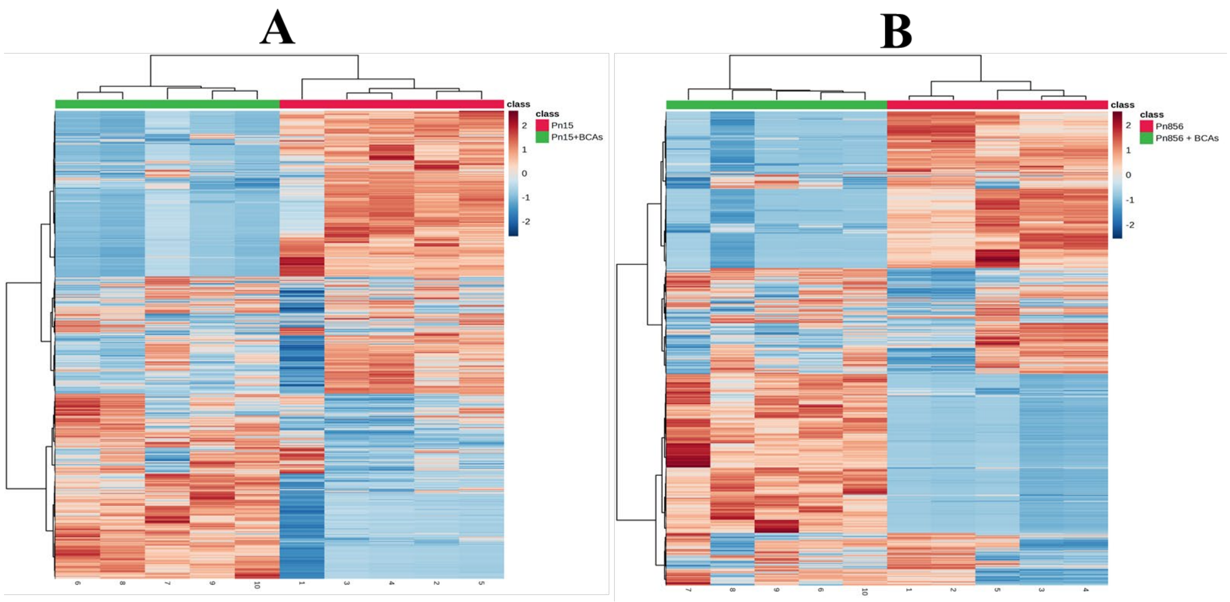

Figure S1. Metabolomic heat maps for *P. nordicum* FHSCC 15 (A) and *P. nordicum* BFE 856 (B) after 30 days in dry-cured ham. Pn15 (red colour): control samples inoculated with *P. nordicum* FHSCC 15; Pn15 + BCAs (green colour): samples inoculated with *P. nordicum* FHSCC 15, *D. hansenii* FHSCC 253H, *S. xylosus* FHSCC Sx8 and *P. chrysogenum* FHSCC Pg222; Pn856 (red colour): control samples inoculated with *P. nordicum* BFE 856; Pn856 + BCAs (green colour): samples inoculated with *P. nordicum* BFE 856, *D. hansenii* FHSCC 253H, *S. xylosus* FHSCC Sx8 and *P. chrysogenum* FHSCC Pg222.

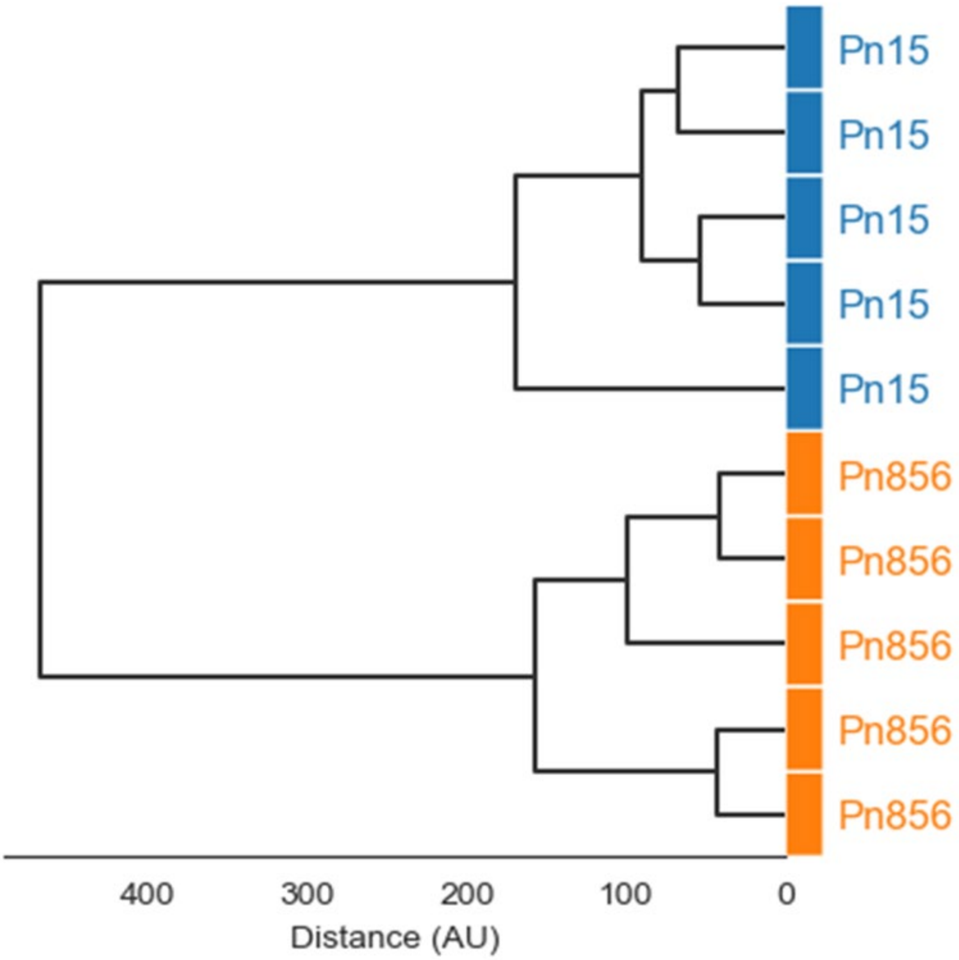

Figure S2. Hierarchical Clustering Analysis (HCA) univariant analysis of metabolome of the batches inoculated with *P. nordicum* FHSCC 15 (Pn15) and *P. nordicum* BFE 856 (Pn856) in dry-cured ham.
